# Simultaneous LC–MS/MS quantification of free deoxyguanosines 2′-dG and 8-OHdG in ovine seminal plasma as biomarkers of oxidative DNA damage

**DOI:** 10.3389/fmed.2026.1824845

**Published:** 2026-05-08

**Authors:** Manuel Alfaro-Gómez, Pedro Javier Soria-Meneses, Julián Garde, María del Rocío Fernández-Santos, Virginia Rodríguez-Robledo

**Affiliations:** 1 Faculty of Pharmacy, University of Castilla-La Mancha, Albacete, Spain; 2 SaBio IREC (CSIC-UCLM-JCCM), Albacete, Spain

**Keywords:** 2′-deoxyguanosine (2′-dG), 8-hydroxy-2′-deoxyguanosine (8-OHdG), LC–MS/MS, oxidative stress biomarkers, seminal plasma, solid-phase extraction (SPE), spermatic DNA

## Abstract

Oxidative stress is harmful for spermatic DNA, since high levels of reactive oxygen species lead to the formation of toxic adducts derived from DNA oxidation. This phenomenon is especially relevant under pathological conditions such as asthenozoospermia, necrozoospermia, oligozoospermia, or azoospermia. The nucleoside most susceptible to this damage is 2′-deoxyguanosine (2′-dG), which is oxidized to form 8-hydroxy-2′-deoxyguanosine (8-OHdG), one of the most widely studied biomarkers of oxidative stress. Previous studies have reported an increased liberation of cell-free DNA during impaired spermatogenesis and cellular necrosis, supporting the presence of free nucleosides in seminal plasma under altered physiological conditions. Consequently, the determination of both biomarkers not only provides information about the oxidative status, but also about cellular turnover. The aim of this study was, therefore, to develop, validate, and apply a reliable and complete analytical methodology for the simultaneous quantification of the mentioned biomarkers in ovine seminal plasma by solid phase extraction (SPE) off-line LC–MS/MS. The procedure includes the study and development of the SPE-based sample preparation protocol, as well as the optimization of instrumental parameters, including the description of the fragmentation pathways for both analytes and the internal standard. Validation followed US FDA guidelines for bioanalytical methods, fulfilling acceptance criteria for precision, linearity response, LOD and LOQ, recoveries, and matrix effect. In addition, sustainability was assessed in accordance with two green analytical chemistry metrics (GAPI and AGREE). The proposed method was applied to seminal plasma extracted from three healthy —pooled sample— and one ram with low sperm quality (LQ)—absence of sperm motility and severely low sperm concentration—. Both 2′-dG and 8-OHdG levels were markedly elevated in the affected ram, with approximately two-fold and four-fold increase, respectively. These promising preliminary results align with the proof-of-concept design of the study. This analytically validated approach represents the first SPE off-line LC–MS/MS method for the simultaneous quantification of free 2′-dG and 8-OHdG in seminal plasma, providing a robust analytical framework for future studies exploring these nucleosides as complementary biomarkers of oxidative DNA with potential applicability to human andrology upon validation in clinical cohorts.

## 1 Introduction

Oxidative stress (OS) is a well-established physiological phenomenon defined as an imbalance between oxidants and antioxidants in favor of the oxidants, leading to disruption of redox signaling control and/or molecular damage (1).

The determination of OS biomarkers *in vitro* and *in vivo* has generated considerable interest for many years, as accurate quantification of this stress is essential to determine its role in certain diseases. Several groups or families of biomarkers can be used to assess OS, including free radicals such as reactive oxygen species (ROS), which are generated through the formation of reactive radicals during cellular metabolism (2, 3). However, these radicals are exceptionally reactive and unstable, with very short half-lives (on the order of seconds), making their analysis highly challenging and, in most cases, limited to qualitative information. To overcome this limitation, quantification of the oxidation products generated by ROS offers a more practical alternative. Endogenously produced ROS can cause damage to proteins, DNA/RNA, and induce lipid peroxidation in cell membranes (4), generating stable oxidation products that serve as reliable biomarkers of oxidative stress.

Among the different biological systems affected by oxidative stress, the male reproductive system is particularly vulnerable. Mammalian sperm cell membranes are rich in polyunsaturated fatty acids (PUFAs), making them particularly vulnerable to free radical attack and peroxidative damage (5). Consequently, OS is increasingly recognized as a key contributor to subfertility and infertility (6). In semen, ROS are produced primarily by leukocytes present in the seminal plasma (SP) or by abnormal and immature sperm. These seminal ROS are natural by-products of oxidative metabolic pathways and are generated by cytosolic and plasma membrane oxidases (7, 8). In this regard, it is important to note that low levels of ROS are produced during normal germ cell metabolism and play crucial regulatory roles in key reproductive processes, including capacitation, acrosomal reaction, hyperactivation, and sperm–ovum fusion (6). In healthy cells, there is an equilibrium between ROS production and the antioxidant defense system (9). However, oxidative stress arises when ROS production surpasses the neutralizing capacity of the antioxidant defense system in seminal plasma. This process has been shown to negatively affect sperm function, causing lipid peroxidation, sperm DNA fragmentation, mitochondrial dysfunction, and germ cell apoptosis (10). DNA damage in sperm has been linked with reduced rates of fertilization, impaired preimplantation development, and miscarriage (11, 12).

Thus, high levels of oxidative stress may damage the spermatic DNA both directly or indirectly through the activation of sperm caspases and the production of endonucleases (10). In the direct pathway, ROS attack nucleic acids —including nuclear and mitochondrial DNA and RNA—, leading to the formation of abundant and relatively stable adducts. Among the DNA bases, guanine is the most susceptible to oxidative attack by ROS due to its low oxidation potential (13). The major DNA lesion associated with adduct generation is 8-hydroxy-2′-deoxyguanosine (8-OHdG), which is formed *in vivo* by oxidation of the nucleoside 2′-deoxyguanosine (2′-dG) (9, 14). This reaction occurs especially in genome regions that are protamine-poor (15). The presence of such adducts weakens the glycosidic bond (7), and since DNA repair capacity in sperm is limited, the enzyme 8-oxoguanine DNA glycosylase 1 (OGG1) generates abasic sites, leading to the release of the oxidized base into the extracellular space, the seminal plasma (7). DNA fragmentation resulting from these processes can compromise cellular integrity and function (6, 16). Because of these observations, 8-OHdG has been established as a reliable biomarker of DNA damage associated with OS.

Given that oxidized bases such as 8-OHdG are released into seminal plasma following DNA damage, this biofluid represents a valuable and minimally invasive matrix for assessing oxidative stress in the male reproductive tract. Seminal plasma is a complex biological medium resulting from the mixture of testicular, epididymal and accessory gland secretions (17). This fluid mediates the chemical function of the ejaculate and plays a crucial role in the regulation of biological processes in sperm (18). Its main components include glycans, lipids, inorganic ions, small-molecule metabolites, cell-free DNA (cfDNA), RNA, microRNAs, peptides, and proteins (19). Although cfDNA and cell-free nucleic acids exist ubiquitously on or outside the cell surface of living organisms (20) their origin in seminal plasma is not fully understood (21). Therefore, several biological processes that may explain their presence have been documented. Although cfDNA was initially thought to be released exclusively by dying cells, evidence suggests it can also be actively secreted by living cells and may serve biological functions in the organism (22). Consequently, the main hypotheses to explain the presence of cfDNA in semen include (i) pathological conditions, such as infections or inflammation, that trigger leukocyte infiltration and neutrophil extracellular trap (NET) formation, contributing to elevated cfDNA concentrations (21, 22); (ii) DNA released by dying cells through apoptosis, necrosis, or NETosis (23); (iii) nucleic acids secreted actively and/or passively by cells of glandular organs—seminal vesicles, prostate, and bulbourethral glands—during cell turnover (21, 24, 25); and (iv) modulation of seminal DNase activity by cations such as Ca^2+^, Mg^2+^, and Zn^2+^ present in seminal plasma (26).

Hence, cfDNA levels in seminal plasma reflect not only cellular turnover but also pathological processes that may be associated with oxidative stress. For instance, in cases of azoospermia, germinal stem cells undergo their differentiation, first through meiosis and then through cellular maturation (21). This process is tightly regulated by checkpoints that can halt differentiation and trigger apoptosis of defective cells. Apoptosis of abnormal cells, particularly aneuploid cells, may therefore increase seminal cfDNA levels (27). Previous studies reported a correlation between cfDNA with significantly higher concentrations in men with azoospermia (21, 22). Furthermore, previous studies mentioned that sperm from boars and mice can internalize exogenous DNA (28), suggesting a potential physiological interaction between sperm and nucleic acids in the seminal environment. Given the abundance of cell-free nucleic acids in seminal plasma and their association with cellular turnover and pathological conditions, detecting and quantifying 2′-dG and 8-OHdG in this biofluid offers a valuable approach to assess oxidative DNA damage in the male reproductive system. These and other oxidized radical-derived products show high stability and have long half-lives (from hours to weeks), making their qualitative and quantitative determination feasible and biologically relevant for monitoring oxidative DNA damage (29).

Since our study was conducted in sheep (*Ovis aries*), an economically important livestock species and a relevant model for reproductive research in mammals, it is important to consider that ovine sperm DNA is highly compacted, a feature that makes the detection of DNA damage by conventional methods challenging. The most widely used methods for detecting sperm DNA fragmentation include sperm chromatin structure analysis (SCSA), performed by flow cytometry; terminal transferase-mediated dUTP end labelling (TUNEL); single-cell gel electrophoresis (SCGE); sperm chromatin diffusion experiment (SCD); and DNA flow cytometry, also known as fluorescence activated cell sorting (FACS) (30). Importantly, none of these techniques is able to detect specific nucleosides such as 2′-dG and 8-OHdG. For this purpose, 2′-dG and/or 8-OHdG quantification has been performed in a range of biological fluids, including tissues (9), urine (14, 31), plasma (32), serum (33, 34), and saliva (35). Various well-established analytical methods have been used for determining these biomarkers in the aforementioned biological matrices. The most commonly employed techniques include enzyme-linked-immune-absorbent-assay (ELISA) (36), gas chromatography coupled to mass spectrometry (GC–MS) (37), high-performance liquid chromatography coupled to electrochemical detection (HPLC-ECD) (35) or to tandem mass spectrometry (LC–MS/MS) (9, 14, 31, 34), and capillary electrophoresis using ultraviolet detection (CE-UV) (38). Nevertheless, despite the large amount of information and the number of methods available for detecting 2′-dG and 8-OHdG, there are no reports of their determination in seminal plasma. Furthermore, although a variety of commercial ELISA kits for measuring 8-OHdG are faster and cheaper than chromatographic methods, it has been demonstrated that anti-8-OHdG antibodies lack the desired specificity, often resulting in overestimation of the 8-OHdG levels (9, 39). Because of these analytical limitations, hybrid separation techniques—specifically liquid chromatography coupled to mass spectrometry (LC–MS/MS)—have emerged as the preferred gold-standard approach for the analysis of oxidative stress biomarkers in biological samples (14). This analytical platform provides high precision, accuracy, sensitivity, and specificity. Furthermore, LC–MS/MS enables simultaneous determination of multiple analytes in complex matrices such as seminal plasma. Moreover, a powerful detector such as a mass spectrometer provides detailed chemical information on the analytes, enabling unequivocal identification, robust quantification, and better interpretation of results (40). In addition, since the methodologies are specifically developed for the quantification of those biomarkers, the development of an LC–MS/MS method for the simultaneous quantification of 2′-dG and 8-OHdG in ovine seminal plasma is clearly warranted.

In addition, determining both nucleosides simultaneously provides more reliable information than the individual determination of 8-OHdG (41). Specifically, as 2′-dG is the non-oxidized nucleoside, its elevation reflects extensive germ cell DNA turnover and a high activity of nucleases, with apoptotic cells failing to complete maturation. Conversely, high levels of 8-OHdG indicate the presence of oxidative stress and DNA damage, primary drivers of apoptosis-like responses in male germ cells, leading to spermiogenesis errors and reduced sperm production (42)Thus, simultaneous quantification of 2′-dG and 8-OHdG enables distinction between different pathophysiological processes—massive cellular turnover, and DNA oxidative damage—in the seminal environment.

Hence, the aim of the study was to develop and validate an analytical method based on a solid-phase extraction (SPE) coupled off-line LC–MS/MS for the simultaneous quantification of 2′-dG and 8-OHdG in ovine seminal plasma, an application not previously reported for this biological matrix. The development process included optimization of chromatographic and mass spectrometric parameters, and characterization of fragmentation pathways for both analytes and the internal standard. Additionally, the environmental sustainability of the method was evaluated using two complementary analytical greenness metrics: the Green Analytical Procedure Index (GAPI) (42) and the Analytical Greenness Calculator (AGREE) (43). Beyond its immediate application in the ovine model, future studies could explore the method’s applicability in human andrology and set the basis for the development of new LC–MS/MS-based methods for the simultaneous quantification of additional biomarkers in seminal plasma.

## 2 Materials and methods

### 2.1 Chemicals and reagents

Standards of the analytes 2′-dG (2′-deoxyguanosine monohydrate, 99–100% purity) and 8-OHdG (8-Hydroxy-2′-deoxyguanosine, ≥98% purity), as well as the internal standard (IS) acetylcholine chloride N, N, N-trimethyl-d9 (≥98% purity) were purchased from Sigma-Aldrich (St. Louis, MO, United States). Formic Acid (98–100% v/v) for LC–MS LiChropur™ was purchased from Merck KGaA (Darmstadt, Germany). Acetonitrile (ACN) LC–MS grade CHROMASOLV TM, methanol for HPLC (≥99.9% purity) and isopropanol CHROMASOLV TM for HPLC (99.9% purity) were supplied by Honeywell (Muskegon, MI, United States). Milli-Q water (18.2 mΩ cm^−1^) for the preparation of the solutions was obtained from a Milli-Q apparatus (Millipore Ibérica S. A., Madrid, Spain).

### 2.2 Standard and stock solutions

A known amount of the analyte 8-OHdG was dissolved in 1 mL of Milli-Q water containing 0,1% (v/v) formic acid (hereafter referred to as H_2_O-FA) to a final concentration of 200 μg mL^−1^. In the case of 2′-dG and the IS acetylcholine-d9 (Ach-d9), were first dissolved in 10 mL of H_2_O-FA to a final concentration of 1,000 μg mL^−1^, and then medium solutions at a 200 μg mL^−1^ were obtained by dilution of each of the stock solutions to a final volume of 1 mL. Working solutions with a concentration of 10 μg mL^−1^ were obtained by dilution of the 200 μg mL^−1^ solutions in H_2_O-FA. All the solutions mentioned were stored at −20 °C. Working solutions were used to prepare the standards and spiked samples. They were also used to prepare mixed working solutions containing both analytes and the IS. Calibration curves were prepared weekly by serial dilution of working solutions.

### 2.3 Sample collection and seminal plasma extraction

Ovine fresh semen was obtained from three adult healthy rams and one ram with low sperm quality (LQ)—absence of sperm motility and severely low sperm concentration—of the Manchega breed (*Ovis aries*), reared on the experimental farm of the University of Castilla–La Mancha. The rams were trained for semen collection using an artificial vagina upon their arrival at the experimental farm. Semen samples are routinely collected and evaluated at least once a week as standard practice. The ejaculates used for this work were collected on three different days over a one-month period. After collection, the samples were transported to the laboratory at room temperature. Immediately thereafter, mass and individual sperm motility (scale 1–5), percentage of motile sperm (%), and sperm concentration (sperm mL^−1^) were evaluated using a bright-field and phase-contrast microscopy (Eclipse 50i Nikon; Tokyo, Japan) (45, 46). Sperm concentration and percentage of motile sperm were assessed using a Makler counting chamber (10 μm depth) at 37 °C, and the Sperm Class Analyzer (SCA^®^) computer-assisted semen analysis (CASA) software (SCA V6.2, Microptic S. L; Barcelona, Spain) (46, 47). A minimum of 5 fields and 300 sperm were analyzed per sample. The image sequences were saved and later analysed. The software settings were adjusted for assessing ram sperm. The parameters used were: 25 frames/s; 30–100 μm^2^ for head area. Samples were then classified into healthy (sample pooled from three rams) and LQ (one ram), based on the evaluation of sperm concentration and motility. Healthy samples were defined as having a minimum wave motion of 4 (subjective assessment) (48), sperm concentrations >4,000 × 10^6^ sperm mL^−1^ and >75% motile sperm. Conversely, the LQ ram presented <150 × 10^6^ sperm mL^−1^ and total absence of motile sperm. It should be noted that the total absence of motile sperm and the extremely low sperm concentration are rare in breeding rams (49), limiting biological and statistical comparisons. However, it is a highly valuable sample to justify the proof-of-concept design of this study. Sperm concentration and motility of each ejaculate are provided in Supplementary Table S1.

For the extraction of the seminal plasma, semen samples were sonicated for 1 min and then centrifuged at 16.500 × *g* at 10 °C for 20 min. The resulting supernatants, constituting the seminal plasma, were pooled—in the case of the healthy group—and aliquoted in 1 mL. Aliquots were subsequently processed for purification and LC–MS/MS analysis or stored at −20 °C until use.

All animal procedures were performed in accordance with the Spanish Animal Protection Regulation RD53/2013, which conforms to European Union Regulation 2010/63/UE.

### 2.4 Sample treatment by solid-phase extraction

A volume of 500 μL of seminal plasma was diluted 1:1 with H_2_O-FA prior to solid-phase extraction (SPE). Isolation and purification were performed using a Visiprep™ SPE vacuum manifold connected to a Millipore vacuum pump. SPE was conducted using Oasis^®^ HLB (6.0 cc, 200 mg) extraction cartridges (Waters, Milford, MA, United States).

The extraction procedure comprised: (i) cartridge conditioning with 4.0 mL ACN followed by 4.0 mL Milli-Q water; (ii) loading of diluted seminal plasma samples (1 mL) at 0.5 mL min^−1^; (iii) cartridge drying under vacuum for 30–60 s; (iv) washing with 4.0 mL Milli-Q water at 1.0 mL min^−1^ to minimize seminal plasma matrix interferences; (v) additional drying for 30–60 s; and (vi) elution with 4.0 mL ACN: Milli-Q water (50:50, v/v) at 0.5 mL min^−1^.

The eluate was collected into test tubes and evaporated with N2 gas. The extracts thus obtained were reconstituted with H_2_O-FA to a final volume of 500 μL. Reconstituted extracts were spiked with the internal standard (Ach-d9) to a final concentration of 250 ng mL^−1^ prior to LC–MS/MS injection. The internal standard was added post-extraction to compensate matrix effects and instrumental variation, as SPE recovery was independently assessed during method validation.

**Figure 1:**
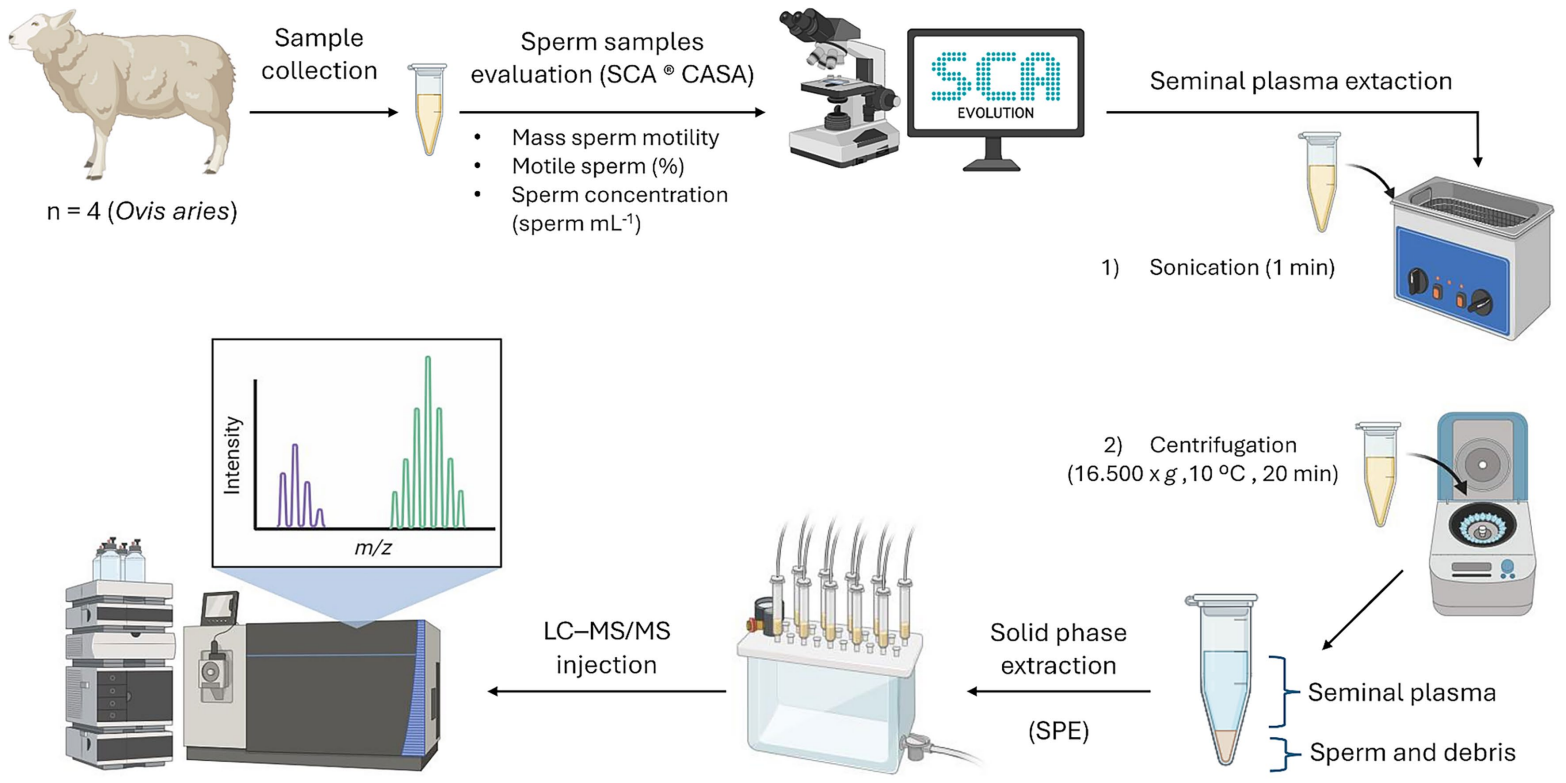
Complete analytical workflow for the simultaneous determination of 2-dG and 8-OHdG in ovine seminal plasma. The process includes semen collection, evaluation of seminal parameters by CASA (SCA® Evolution), seminal plasma extraction, sample treatment by off-line solid-phase extraction (SPE), and LC–MS/MS analysis.

A schematic overview of the full process, from sample collection to LC–MS/MS injection, is provided in Figure 1.

### 2.5 Liquid chromatography and mass spectrometry conditions

Analytes were separated on an Agilent 1260 Infinity HPLC system equipped with a binary pump and vacuum degasser. Samples were injected with an Agilent autosampler fitted with a 25 μL needle and analyzed on an Agilent 6460 triple quadrupole (QqQ) mass spectrometer (Agilent Technologies, Santa Clara, CA, United States) equipped with an electrospray (ESI) interface. Zero-grade nitrogen used for the ESI source and QqQ detector was supplied by a nitrogen generator from PEAK Scientific (Chicago, IL, United States).

Chromatographic separation was performed on a ZORBAX Eclipse XDB-C18 column (4.6 mm × 250 mm, 3.5 μm) at 25 °C with a guard column and using gradient elution at 0.4 mL min^−1^. The aqueous mobile phase (A) consisted of Milli-Q water with 0.1% (v/v) formic acid (H_2_O-FA), while the organic mobile phase (B) was ACN with 0,1% (v/v) formic acid. The elution program was as follows: 30% B for 2 min, decreased to 10% B over 1 min and held for 3 min, then returned to 30% B over 1 min with a 1 min hold. The injection volume was 10 μL for both standards and samples.

The ESI source operating conditions were as follows: drying gas temperature of 300 °C, drying gas flow rate of 11 L min^−1^, nebulizer pressure of 30 psi, and a capillary voltage of 3,000 V. Analytes were detected and quantified using multiple reaction monitoring (MRM) in positive ionization mode, employing the acquisition parameters summarized in Table 1. For each analyte, at least two MRM transitions were monitored: the most abundant transition was used for quantification, and the secondary transition(s) for qualitative analysis and confirmation.

**Table 1:** MS/MS acquisition parameters.

Analytes and IS	Molecular weight (g mol^−1^) and formula	Precursor ions (m/z)	Product ions (m/z)	Fragmentor (V)	Collision energy (eV)
2′-dG	267,24 C_10_H_13_N_5_O_4_	268.0	152.0*	115	18
			135.0	135	37
8-OHdG	283.24 C_10_H_13_N_5_O_5_	284.1	168.1*	110	14
			140.1	130	28
Ach-d9 (IS)	155.21 C_7_H_16_NO_2_^+^	155.2	87.0*	93	16
			69.2	93	8
			43.2	93	32

*Quantitative ion for each analyte.

### 2.6 Validation procedure and data analysis

The reliability of the method for 2′-dG and 8-OHdG determination was established by using triplicate injections to assess signal intensity, relative peak area (RPA), and signal-to-noise (S/N) ratio. A systematic validation of the LC-ESI-MS/MS chromatography method, which followed the US FDA guidelines for bioanalytical methods was carried out. The method was evaluated in terms of precision, linearity response, limit of detection (LOD) and limit of quantification (LOQ). Regarding the sample treatment process, recoveries and matrix effects were tested.

MassHunter Quantitative Analysis B.09.00 and MassHunter Qualitative Analysis Navigator B08.00 software provided by Agilent Technologies (Santa Clara, CA, United States) were used to process the chromatographic results. All statistical treatments were performed by Microsoft Excel® (Microsoft Corporation, Redmond, WA, United States) software.

## 3 Results and discussion

### 3.1 Development of the analytical method

The operational chromatographic and mass spectrometry conditions were optimized to achieve maximum signal intensity, optimal peak shape, and signal stability. Although baseline separation of analytes is not critical when using selective MRM detection, short retention and analysis times were prioritized to minimize solvent consumption and comply with green analytical chemistry principles.

Consequently, chromatographic conditions for the simultaneous separation of 2′-dG and 8-OHdG by LC–MS/MS were adapted from methods previously published (9, 14). Initial conditions comprised reversed-phase LC using a C18 column (2.1 mm × 50 mm, 1.8 μm) at 25 °C with a guard column, and isocratic elution at 0.3 mL min^−1^. Mobile phase consisted of H_2_O-FA (A) and ACN (B) at 80:20 (v/v). Under these conditions, analytes exhibited poor retention on the stationary phase, presumably due to their relatively high polarity. The proportion of the aqueous phase was increased to enhance analytes retention through the stationary phase; however, this resulted in excessive backpressure that exceeded instrumental limits. Although gradient optimization could partially address retention issues with the shorter column, a longer column with larger particles was preferred to accommodate the high aqueous content required for these polar analytes while maintaining acceptable backpressure and avoiding excessively steep gradients. To overcome this limitation, columns with larger particle size were evaluated. Among those tested, the ZORBAX Eclipse XDB-C18 column (4.6 mm × 250 mm, 3.5 μm) with guard column proved most suitable, providing stronger analyte retention, improved peak shape, and higher chromatographic efficiency at lower backpressure. This C18 stationary phase operates in reverse mode but allows a very low proportion or even absence of organic solvent, thereby enhancing retention and separation of the polar analytes. The final optimized method, including gradient elution, composition, and proportion of mobile phase, was described in Section 2.5. These preliminary evaluations established the chromatographic foundation for subsequent fine-tuning of LC parameters and optimization of MS/MS conditions.

Further sub-sections describe the optimization of the solid-phase extraction procedure and the selection of mass spectrometry parameters.

#### 3.1.1 Optimization and selection of liquid chromatography conditions

Following selection of the ZORBAX XDB-C18 column (Section 3.1.2), systematic optimization of mobile phase composition, elution mode, and flow rate was performed to maximize analyte signal, improve peak shape, and minimize analysis time.

The isocratic elution with H₂O-FA and varying proportions (40–80%) of acetonitrile, methanol, or isopropanol resulted in poor peak resolution, overlapping signals, and inadequate analysis times. Acetonitrile was ultimately selected as the organic component due to superior peak shape and signal intensity compared to methanol and isopropanol. Thus, both aqueous and organic mobile phases contained 0.1% (v/v) formic acid. Under isocratic conditions, retention times were excessively long; gradient elution was therefore implemented to improve efficiency. Several gradient profiles were evaluated, and the optimal program, described in Section 2.5, provided the best compromise between resolution and analysis time.

The influence of the mobile phase flow rate was examined over the range 0.2–0.6 mL min^−1^. A flow-rate of 0.4 mL min^−1^ was deemed optimal in terms of peak shape and retention times, enabling separation of both analytes and the internal standard within 8 min. Column temperature was maintained at 25 °C, as no significant differences were observed at other temperatures.

Finally, the injection volume was optimized, as this parameter is critical when analyzing biological samples. An increased injected volume is expected to introduce greater amounts of biological components into the system, potentially causing stronger interferences of the biological matrix with the column and impairing analyte separation. By contrast, very low injected volumes could lead to scarcely reproducible results, subject to large errors (50). For this method, 10 μL was selected as the optimal compromise between sensitivity and reproducibility.

With chromatographic parameters fully optimized, attention was directed toward selection and optimization of mass spectrometry conditions.

#### 3.1.2 Study and selection of ESI-MS/MS parameters

The main purpose of optimizing the ESI-MS/MS variables was to ensure stable, reproducible, and selective signals. To this end, the influence of ionization-related parameters—including nebulizer flow-rate, temperature, and pressure—was evaluated, as well as precursor ion selection, product ions, and transition optimization.

Initially, the most abundant precursor ion from each analyte was selected based on full-scan mode analysis. Specific precursor ion-related variables, including transitions, fragmentor voltage (V), and collision energy (eV) were selected by using the Optimizer function in Agilent MassHunter software. Acquisition parameters for each analyte and the IS are summarized in Table 1. Due to the difficulties in acquiring commercially available deuterated standards for these analytes, their extremely high cost, and limited stability, several deuterated analogous molecules were evaluated. To overcome these limitations, acetylcholine-d9 was selected as the internal standard based on its similar ionization behavior under the same ESI conditions and retention times within the analytical window. Although the use of a non-isotopically matched IS not standard practice, acetylcholine-d9 provided reliable correction of matrix effects, recovery, and signal variability throughout the analytical sequence, as detailed within the validation Section 3.2. For all analytes, ionization was more stable and sensitive in positive ion mode [M + H]^+^ than in negative ion mode [M–H]^−^.

**Figure 2:**
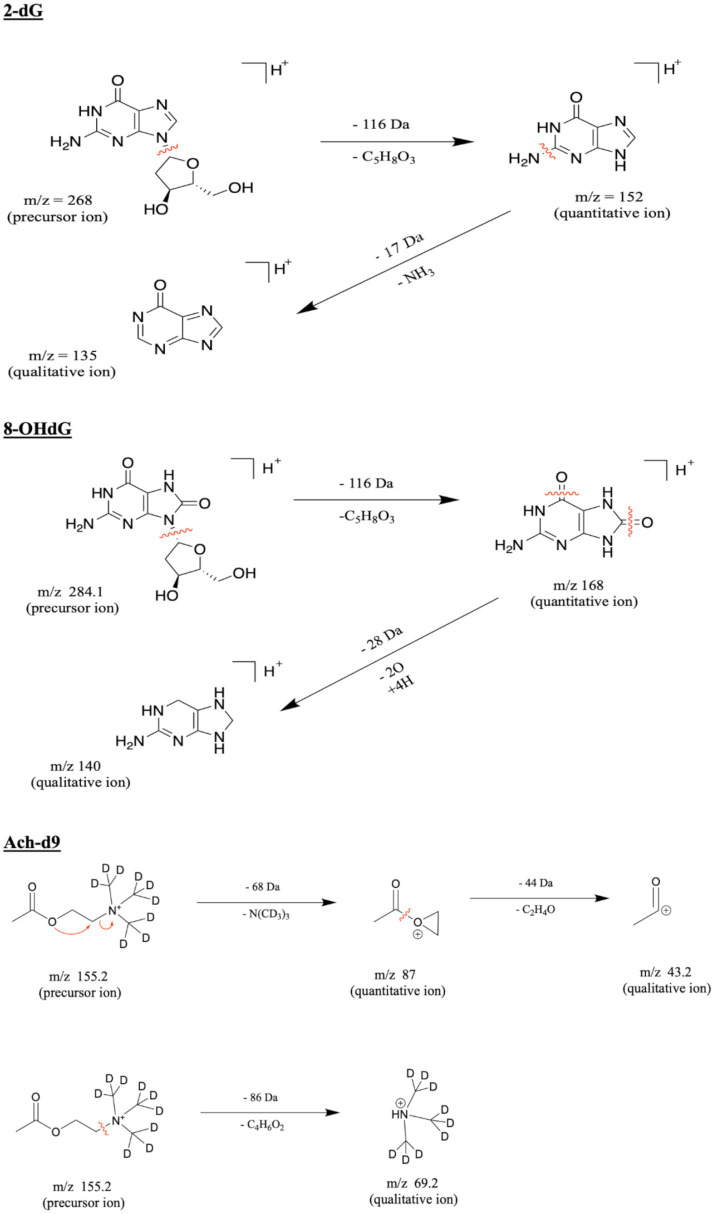
Proposed fragmentation pathways for 2′-dG, 8-OHdG, and the internal standard, respectively.

To our knowledge, the fragmentation pathways corresponding to the analytes have not been previously reported. The proposed fragmentation routes and product ion assignment from the precursor ions of 2′-dG, 8-OHdG, and the IS are presented in Figure 2.

The highest transitions of each precursor ion were used for quantification, and the remainder for qualitative identification. Operational variables such as drying gas flow rate, gas temperature, and nebulizer pressure —which directly impact the ionization process— were systematically examined over the ranges afforded by the instrument: 5–13 L min^−1^, 250–350 °C, and 10–60 psi, respectively. The selected conditions, which provided optimal results in terms of RPA, stable signals, and S/N ratio, are described in Section 2.5.

Analytes were detected and quantified using positive ion polarity in multiple reaction monitoring (MRM) mode. Method development was supported by total ion chromatograms (TIC) obtained in full-scan mode and evaluation of selected transitions for each compound.

Supplementary Figure S3 shows a representative LC–MS/MS chromatogram of a seminal plasma sample obtained during method optimization, illustrating the simultaneous and baseline-resolved detection of both 2′-dG and 8-OHdG under the optimized chromatographic conditions.

A representative LC–MS/MS chromatogram of a seminal plasma sample obtained during method optimization, illustrating the simultaneous detection of both 2′-dG and 8-OHdG under the optimized conditions, —and their molecular structures— is provided in Supplementary Figure S1.

#### 3.1.3 Study of the solid-phase extraction

As previously mentioned, seminal plasma is a complex biological matrix. Moreover, in contrast to other biological fluids, SP is an alkaline medium in which several compounds are ionized. This large variety of components can interfere with the determination of the target analytes, increasing the matrix effect. For this reason, an in-depth study of the sample treatment required for the biological sample was needed. Such treatment was carried out by solid-phase extraction. The main objectives of SPE were to remove as many interfering components as possible—thereby reducing matrix effects—, and to facilitate the isolation of the analytes. However, SPE is a complex procedure since it may produce the loss of a considerable proportion of the target analytes alongside the interferents.

Seminal plasma samples were obtained from fresh ovine semen as described in Section 2.3. Prior to SPE, 500 μL aliquots were diluted 1:1 with H_2_O-FA to acidify the seminal plasma, thus favoring the neutral form of the analytes and facilitating their retention on the Oasis^®^ HLB extraction cartridges. The influence of the experimental variables (viz., nature and volume of the organic and aqueous solvents used in the washing steps, sample volume, eluted volume, and final extract volume, among others) was examined after each step of the SPE process.

Because the HPLC column used for analysis was C18-based, extraction cartridges with the same composition were initially evaluated. Under these conditions, they exhibited poor retention capacity, likely due to their highly nonpolar characteristics. Subsequent evaluations incorporated cartridges with higher polarity (−2OH), but these also proved unsuitable. Finally, cartridges of intermediate polarity—specifically Oasis^®^ HLB cartridges (6.0 cc, 200 mg)—were selected and proved most suitable for this application.

The critical steps of the SPE were carefully optimized, including the volume and composition of the washing and elution solutions, as well as the vacuum pressure applied at each step. The final optimized procedure is described in Section 2.4.

### 3.2 Method validation

For the evaluation of the validation parameters, signal intensity, RPA, and S/N ratio from triplicate injections of each analyte and the internal standard were used. These were obtained by monitoring the mass-to-charge ratio ions of m/z [152.0]^+^, [168.1]^+^, and [87.0]^+^ for 2′-dG, 8-OHdG, and IS (Ach-d9), respectively. Chromatographic peaks were integrated automatically using valley-to-valley integration and subsequently verified manually to ensure accuracy.

Intra-day precision was evaluated from retention time (RT) and relative peak areas (RPA) for each analyte and the IS by replicate analysis (*n* = 15). Concentrations were set at 10 ng mL^−1^ for the analytes and 500 ng mL^−1^ for the IS. Relative standard deviation (RSD) values <5% were obtained for both RT and RPA. Inter-day reproducibility was assessed using Snedecor’s *F*-test through sequential injection of 15 freshly prepared samples under identical conditions at 48-h intervals over 15 days. No statistically significant differences between variances (S^2^) were observed.

The calibration curves used for the evaluation of the linearity range corresponded to spiked ovine seminal plasma at 7 concentration levels ranging from 10 to 750 ng mL^−1^. This range was selected to encompass the expected physiological concentrations in ovine seminal plasma, which were estimated from preliminary experiments. Adequate linearity with *R*^2^ values over 0.9995 was obtained for both analytes. To assess the matrix effect, two calibration curves at the same concentration range were compared: one corresponding to standard solutions and the other to spiked ovine seminal plasma (Supplementary Figure S2). Matrix effects, calculated as the ratio of slopes (slope matrix/slope standard solutions), were 96.20% for 2′-dG and 102.40% for 8-OHdG, indicating irrelevant ion suppression or enhancement, within the acceptable range of 85–115%. One-way ANOVA confirmed no significant differences between the two curves. These results support the acetylcholine-d9 suitability as internal standard despite not being isotopically matched, since it provided adequate correction for matrix-induced signal variability. All calibration curves, including calibration curve equations, are represented in Supplementary Figure S3.

LOD LOQ were experimentally calculated for both 2′-dG and 8-OHdG, obtaining values of 0.7 and 2.5 ng mL^−1^ for 2′-dG, while 0.05 and 0.17 ng mL^−1^ for 8-OHdG, respectively.

Recovery of the SPE process was evaluated at two concentration levels (250 and 750 ng mL^−1^) by comparing spiked seminal plasma with standard solutions (*n* = 3 injections per level). Mean recoveries were over 80% for both concentrations of 2′-dG and 8-OHdG, confirming the accuracy of the method.

### 3.3 Greenness evaluation

Currently, in addition to the development of an instrumental methodology with a high analytical quality that allows adequate interpretation of the results from a physiological or biochemical perspective, the environmental and sustainable aspects of the method are of great importance. For this reason, the green aspect was evaluated using two analytical metrics, Green Analytical Procedure Index (GAPI) (42) and Green Analytical Greenness Calculator (AGREE) (43).

The GAPI tool uses a color-coded scheme—green (low environmental impact), yellow (medium environmental impact), and red (high environmental impact)—to intuitively express results, considering aspects from sampling, sample preparation, solvents/reagents, and analytical instrumentation. AGREE complements this evaluation by providing a numerical score ranging from 0 to 1, with values closer to 1.0 indicating excellent greenness (51).

**Figure 3:**
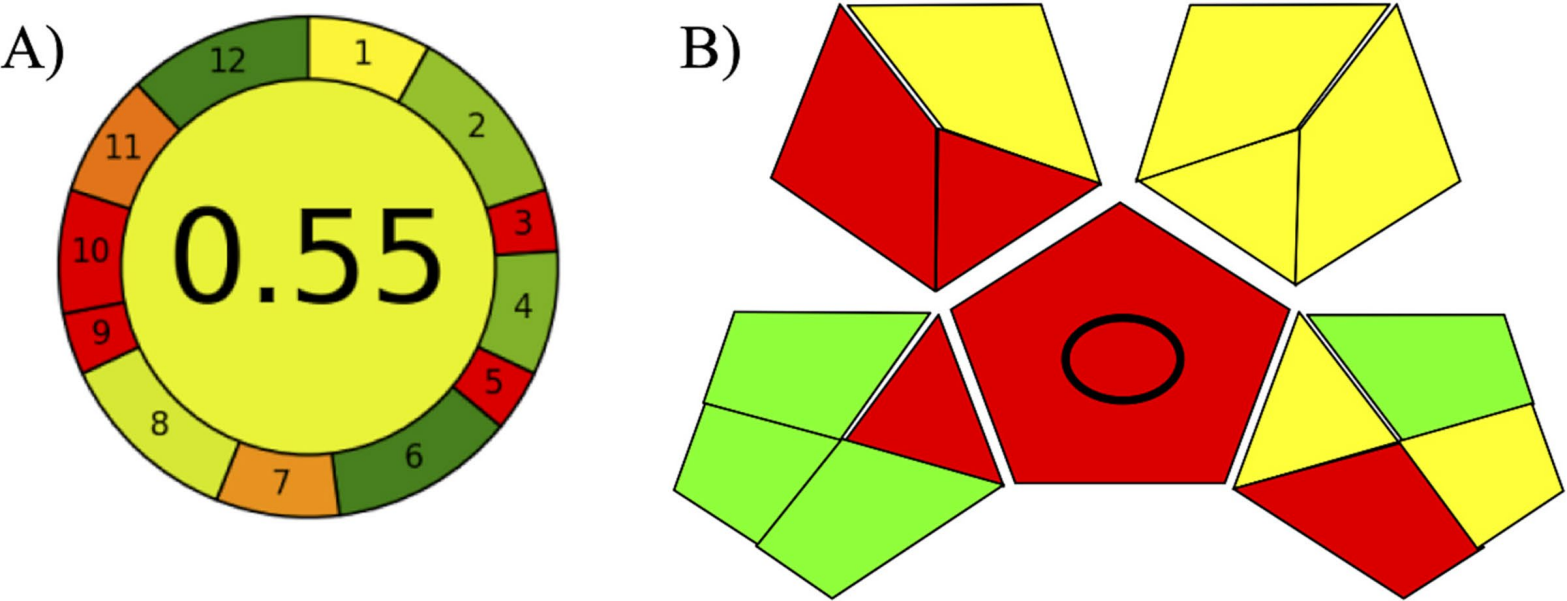
Result of the green evaluation with of the proposed analytical methodology for the determination of 2′-dG and 8-OHdG in seminal plasma samples. **(A)**: AGREE, **(B)**: GAPI.

According to the greenness evaluation results (Figure 3), the developed methodology exhibits a reasonable green character. The main penalties are related to off-line sampling, a characteristic which is virtually unavoidable on seminal plasma analysis in research laboratories, and the use of formic acid and acetonitrile, although only small volumes are necessary. As positive aspects, the method does not involve a derivatization procedure, requires only 500 μL of biological sample, and presents minimal risk to the analyst. Furthermore, this method enables the simultaneous determination of two analytes, using 8 min per analysis. The application of LC–MS/MS, while representing a penalty in greenness metrics due to solvent consumption and energy requirements, remains essential for achieving the required sensitivity and specificity. All factors mentioned above were considered when building the AGREE index, with emphasis on parameters susceptible to modification. Greenness evaluation is, therefore, essential as environmental sustainability is a key feature of modern analytical methods.

### 3.4 Application of the proposed method to seminal plasma samples

As a first biological application, the developed methodology was applied to ovine (*Ovis aries*) seminal plasma to compare 2′-dG and 8-OHdG levels between samples obtained from 3 different healthy rams (subsequently pooled), and samples obtained from the LQ ram. All ejaculates were collected and classified by routine semen analysis as described in Section 2.3. Three ejaculates per group were obtained on different days, and seminal plasma was extracted for individual analysis by SPE off-line LC–MS/MS using the developed method. Each sample was injected three times. Results are expressed as mean concentrations ± SD of nine injections per group (three samples × three injections each), to assess technical variability. The intra-sample RSD was below 10% in all cases. No statistical comparison between healthy and LQ was intended or performed, as the biological application serves to demonstrate the applicability of the method within the framework of this methodological study. These results are shown in Table 2.

**Table 2:** Concentrations of 2′-dG and 8-OHdG in seminal plasma by group, with intra-sample RSD (%).

Group	[2′-dG] ng mL^−1^				[8-OHdG] ng mL^−1^			
	Mean ± SD (3 ejaculates × 3 injections each)	Intra-sample %RSD			Mean ± SD (3 ejaculates × 3 injections each)	Intra-sample % RSD		
		R1	R2	R3		R1	R2	R3
Healthy (*n* = 3 rams)	10.58 ± 1.94	6.99	1.51	3.44	4.73 ± 0.40	5.66	4.71	0.83
LQ (*n* = 1 ram)	21.27 ± 3.35	4.19	2.96	3.60	16.90 ± 6.78	8.55	4.03	7.55

Results are expressed as mean ± SD of 9 injections (3 ejaculates per group, each injected 3 times). R1, R2, R3 refer to individual ejaculate replicates (technical replicates).

**Figure 4:**
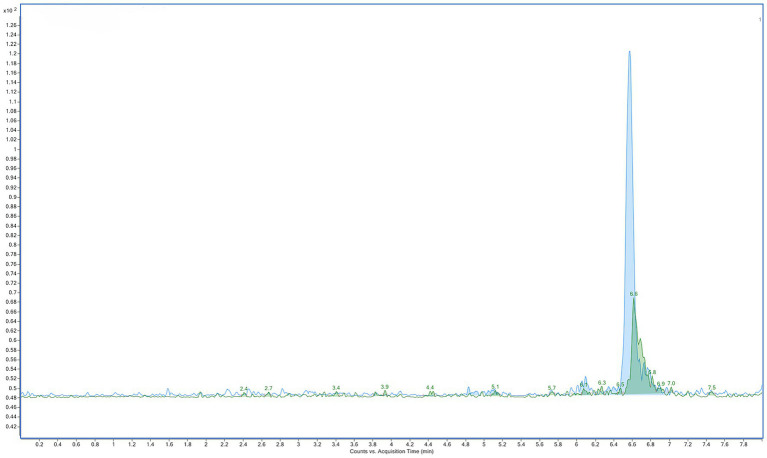
Representative LC–MS/MS chromatograms showing 2′-deoxyguanosine (2′-dG) levels in seminal plasma. The 2′-dG MRM signal is shown for the healthy pooled sample (green) and the LQ sample (blue).

Therefore, the analyses showed that both 2′-dG and 8-OHdG levels were elevated in the LQ ram, with 2′-dG exhibiting approximately a two-fold increase compared to an almost four-fold increase for 8-OHdG. The elevation in 2′-dG was further illustrated by representative chromatograms (Figure 4), in which the 2′-dG signal was substantially higher in the LQ sample compared to the healthy pooled seminal plasma. These findings are consistent with previously reported studies in similar pathological conditions affecting male fertility (21, 22). Although further studies with larger cohorts are required (21, 51), these preliminary results, framed as proof-of-concept, support the applicability of the LC–MS/MS method for quantifying these biomarkers to assess pathophysiological changes in the male genital tract.

## 4 Conclusion and future perspectives

Overall, this study reports the first LC–MS/MS method for the simultaneous determination of free 2′-dG and 8-OHdG in seminal plasma. Validated according to FDA bioanalytical guidelines, the developed method combines solid-phase extraction (SPE) with highly specific LC–MS/MS detection, enabling robust and precise quantification of both oxidized and non-oxidized guanosine nucleosides within a single analytical run. Its application to ovine seminal plasma yielded preliminary results consistent with prior studies, with the main contribution of this work lying in the method development and validation. These may provide insight into the specific nucleosides present in seminal plasma under physiological and pathological conditions, where oxidative DNA damage is hypothesized to increase.

This approach offers, a robust framework for subsequent studies providing high accuracy, sensitivity, selectivity, and reproducibility. Beyond its application in the ovine model, which is highly relevant in reproductive physiology and andrology, it may enable future investigations exploring seminal plasma 2′-dG and 8-OHdG as valuable biomarkers of oxidative stress and DNA damage, with potential future application pending validation in human cohorts.

## Data Availability

The raw data supporting the conclusions of this article will be made available by the authors, without undue reservation.
