# Supplementary material for: Simultaneous LC–MS/MS quantification of free deoxyguanosines 2′-dG and 8-OHdG in ovine seminal plasma as biomarkers of oxidative DNA damage
Source: Front Med (Lausanne). 2026 May 8;13:1824845. doi: 10.3389/fmed.2026.1824845 (PMC13194507; doi:10.3389/fmed.2026.1824845)
Supplement: Supplementary file 1 [file Data_Sheet_1.docx]

**Table S1.** Semen parameters of healthy and low-quality sperm (LQ) ram ejaculates

| Sample | Sperm concentration | percentage of motile sperm |
| --- | --- | --- |
| LQ (I) | 110 x 10^6^ sperm mL^-1^ | 0 % |
| LQ (II) | 68 x 10^6^ sperm mL^-1^ | 0 % |
| LQ (III) | 32 x 10^6^ sperm mL^-1^ | 0 % |
| Healthy (I) | 4900 x 10^6^ sperm mL^-1^ | 78 % |
| Healthy (II) | 9471 x 10^6^ sperm mL^-1^ | 88,1 % |
| Healthy (III) | 6507 x 10^6^ sperm mL^-1^ | 88,3 % |

**Supplementary Figure 1**

8-OHdG

**2’-dG**

**8-OHdG**

**Figure S1.** Representative LC–MS/MS chromatogram of a seminal plasma sample obtained during method optimization. Green peak corresponds to 2’-dG (RT = 6.6 min), while the blue one corresponds to 8-OHdG (RT=6.8 min). Chemical structures of both biomarkers are also represented.

**Supplementary Figure 2**

**A**

**B**

**Supplementary Figure 2.** Comparison of the calibration curve ranged among 10.0 and 750.0 ng mL^–1^ for 2’-dG **(A)** and 8-OHdG **(B)**. Black points correspond to the calibration curve carried out with working solutions, while pink triangles represent the calibration curve obtained with spiked ovine seminal plasma samples.

**Supplementary Figure 3**

**A**

**B**

**C**

**D**

**Supplementary Figure 3.** Calibration curves for 2′-deoxyguanosine (2-dG) and 8-hydroxy-2′-deoxyguanosine (8-OHdG) in working solutions and spiked seminal plasma. Linear calibration curves obtained for 2-dG **(A)** and 8-OHdG **(B)** in working solutions. Calibration curves obtained for 2-dG **(C)** and 8-OHdG **(D)** in spiked ovine seminal plasma.Calibration curve equations and coefficients of determination (R²) are shown for each curve.
